# Biplane Imaging Using Portable Ultrasound Devices for Vascular Access

**DOI:** 10.7759/cureus.12561

**Published:** 2021-01-07

**Authors:** David Convissar, Edward A Bittner, Marvin G Chang

**Affiliations:** 1 Anesthesiology and Critical Care, Massachusetts General Hospital, Boston, USA; 2 Anesthesia, Critical Care, and Pain Medicine, Massachusetts General Hospital, Boston, USA

**Keywords:** biplane imaging, multiple plane, ultrasound, xplane imaging, vascular access, biplane, critical care, xpane, pocus, arterial line

## Abstract

The use of ultrasound guidance for the placement of difficult IVs, arterial lines, and central venous access has become the standard of care. While imaging quality has improved over the last two decades, the lack of affordability, availability, and training have been major limitations in its routine clinical use. We detail the first reported use of biplane imaging using a portable ultrasound probe for difficult vascular access to increase first past success, efficiency, safety, and sterility during the coronavirus disease 2019 (COVID-19) pandemic.

## Introduction

The use of ultrasound for obtaining vascular access is common practice in the intensive care unit (ICU) [1]. Due to a plethora of factors associated with a critical illness, the need for a variety of arterial and venous access is vital for optimal care of many patients. While access can often be obtained by palpation or visual inspection in non-critically ill patients, obstacles such as anasarca, the use of vasoactive agents and more make the use of ultrasound even more important in the ICU [1, 2]. Until recently, the use of bedside ultrasound for the placement of arterial, central and peripheral lines has been limited to single plane imaging. This means that the practitioner may only view a vessel in either the short or long axis at any given time. The use of biplane ultrasound imaging allows the practitioner to view a vessel simultaneously in the short and long axis while performing vascular access procedures, which may increase procedural success, efficiency, and safety.

While biplane imaging (also referred to as Xplane) is regularly used by cardiac anesthesiologists and cardiologists to interrogate cardiac structures and devices, the technology has not been routinely used for vascular access [3, 4]. Here, we report the first use of biplane imaging using an inexpensive ($1,999) portable ultrasound probe for difficult vascular access to increase first past success, efficiency, safety, and sterility during the coronavirus disease 2019 (COVID-19) pandemic.

## Case presentation

An 83-year-old man with a medical history significant for obesity, hypertension, hyperlipidemia, type II diabetes, and chronic obstructive pulmonary disease (COPD) presented to the emergency department (ED) with acute hypoxemic respiratory failure secondary to COVID-19 pneumonia. The patient was intubated and subsequently developed severe hypotension requiring support with high dose vasopressor agents. To facilitate continuous blood pressure and arterial blood gas monitoring, radial artery cannulation was attempted multiple times without success.** **Initially, cannulation was attempted blindly and then with out-of-plane ultrasound guidance.

**Figure 1:**
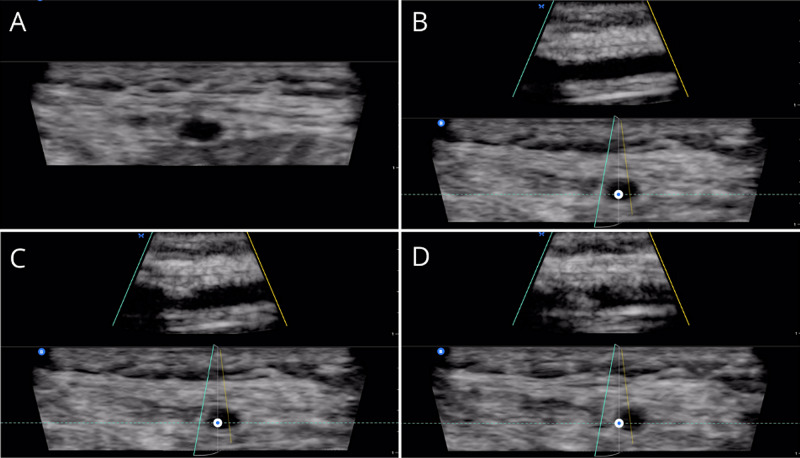
Views of the radial artery during sequential stages of arterial line access and insertion with the Butterfly IQ+ handheld ultrasound device A: The vessel in short-axis view. B: The vessel in biplane view, both long and short. C: The vessel in biplane view with needle entering the vessel. D: The vessel in biplane view with a needle within the vessel.

The intensive care unit (ICU) fellow was called to provide assistance and evaluate the patient in the ED. On the assessment of the right radial artery using biplane imaging with the Butterfly IQ+ (Butterfly Network, Inc., Guilford, CT) handheld ultrasound device attached to his cell phone (iPhone 11 Pro-Butterfly iQ app) as previously described [5], it was found to be small (0.2cm) and approximately 0.75cm deep to the skin with a small luminal diameter, subjectively weak pulsatility and easy collapsibility with compression. The patient’s wrist was prepped and draped in a sterile fashion and positioned with a radial wrist bump. The Butterfly IQ+ ultrasound device was set to “vascular access” and the “biplane” mode was selected. A micro-puncture needle and wire (Cook G0339 Micropuncture® Introducer Set, Bloomington, IN) were used to obtain access to the artery. The needle was able to be visualized within the lumen of the artery in both the long and short axis simultaneously, and the back wall of the vessel was not violated (Figure 1). A guidewire was threaded and visualized in both the long and short axis views without arterial wall dissection, and the catheter was threaded and secured. Only one attempt was made for successful placement, and there were no complications with the procedure. The patient was subsequently transferred to the ICU. 

## Discussion

This case highlights not only the importance of portable ultrasound technology but the utility of the biplane view for difficult vascular access. Due to the critical state of the patient obtaining arterial access was extremely challenging, as noted by the number of attempts taken and failure to cannulate even with standard ultrasound assistance prior to the ICU’s intervention. In the setting of rapid clinical deterioration of a patient, rapid access to a portable ultrasound device on hand may be instrumental in providing swift and appropriate patient care such as arterial access for pressure management, central line access for pressors, and more [6, 7]. Because of their compact nature, ease of use and cleaning (no buttons, nooks and crannies), these portable devices help to decrease viral spread, which is crucial during the COVID-19 pandemic [5]. Unlike traditional ultrasound machines, which are large and cumbersome to move, these highly portable devices are more readily and easily utilized in non-ideal circumstances such as under the drapes in the operating room or in a busy ED during acute resuscitation of a patient. Such a device carried in the back pocket can be rapidly deployed within seconds for urgent vascular access and point of care ultrasound evaluation. 

Vascular access in the critically ill patient is particularly challenging due to the patient’s underlying disease process as well as the clinical urgency for management. Failed attempts at vascular access (both venous and arterial) are not uncommon in this patient population, even with skilled practitioners utilizing standard ultrasound [8, 9]. The use of biplane technology may further enhance visualization during vascular access, thereby potentially improving first pass success and safety in this particularly vulnerable patient population. We acknowledge that the use of a micropuncture kit in addition to biplane imaging could have contributed to the success of obtaining vascular access in our patient. Furthermore, the learning curve for vascular access may be much lower with biplane imaging as it does not require advancing the ultrasound probe with the needle tip for successful vascular access, a technique which may not be readily intuitive to many practitioners who are attempting vascular access using ultrasound technology for the first time. Biplane imaging technology may improve patient safety by reducing excessive needle advancement, thereby decreasing the risk of complications associated with both simple and more complex vascular access procedures such as inadvertent carotid puncture, pseudoaneurysm formation, and pneumothorax [10, 11]. Its use may also be applicable to regional procedures such as nerve blocks, where it may also decrease the risk for local anesthetic systemic toxicity (LAST) due to unintentional intravascular injection, which can result in cardiovascular collapse [12]. While this may be the result of user or technique error, the advanced imaging may help to mitigate the consequences of improper or novice technique.

Given the easy portability, sterility, affordability, and integration of innovative technologies such as biplane imaging and teleguidance [13], we anticipate that handheld devices will become commonplace for performing vascular access in the most sterile, quick, efficient, and safe manner by practitioners of different subspecialties and skill levels.

## Conclusions

Ultrasound-guided vascular access is becoming more commonplace, from simple IV insertion and arterial/central line placement to complex percutaneous extracorporeal membrane oxygenation (ECMO) cannulation. The need for improved ultrasound technology to ensure ease of use and safety is therefore increasingly important. Biplane imaging has already proven crucial in the evaluations of cardiac valves, and the translation of that technology to vascular access could greatly improve safety and efficacy with ultrasound-guided vascular access. 
